# Feasibility, safety, and efficacy of the amber peripheral liquid embolic system: results from stage I of the first-in-human OPAL study

**DOI:** 10.1186/s42155-026-00797-2

**Published:** 2026-11-11

**Authors:** Elena Serrano, José Urbano, Javier Irurzun, Mercedes Pérez, Iñigo Insausti, Raúl García, Arantxa Gelabert, Serena Pisoni, Roberto Correa, Andrés Guirola, Miguel Cibrán, Juanjo Espejo, Mariano Magallanes, Sara Pich, Aina Fernández, Sofia Valle, Álex Gómez, María Molina, Antonio López-Rueda, Jose Joaquín Martínez, Lluis Duocastella, Fernando M. Gómez, research team of the OPAL study

**Affiliations:** 1 Department of Interventional Radiology, Hospital Universitario de Bellvitge, Hospitalet de Llobregat, Barcelona, Spain; 2 Department of Interventional Radiology, Hospital Ramón y Cajal, Madrid, Spain; 3 Department of Interventional Radiology, Hospital General Universitario Dr Balmis, Alicante, Spain; 4 Department of Interventional Radiology, Hospital Universitari Vall d’Hebron, Barcelona, Spain; 5 Department of Interventional Radiology, Hospital Universitario de Navarra, Pamplona, Barcelona, Spain; 6 Department of Interventional Radiology, Hospital Universitari I Politècnic La Fe Hospital, Valencia, Spain; 7 Department of Interventional Radiology, Hospital Clinic de Barcelona, Barcelona, Spain; 8 Department of Interventional Radiology, Hospital Universitari I Politècnic La Fe Hospital, Data Manager, Valencia, Spain; 9 Department of Interventional Radiology, Hospital Universitari Parc Taulí, Sabadell, Barcelona, Spain; 10 Department of Interventional Radiology, Hospital Lozano Blesa, Saragossa, Spain; 11 Department of Interventional Radiology, Interventional Nursing, Hospital Universitari I Politècnic La Fe Hospital, Valencia, Spain; 12 Department of Interventional Radiology, Hospital Universitario Reina Sofía, Córdoba, Spain; 13 Department of Interventional Radiology, Hospital Ribera Povisa, Vigo, Spain; 14 iVascular S.L.U., Sant Vicenç Dels Horts, Barcelona, Spain

**Keywords:** Embolization, Therapeutic, Interventional radiology, Endovascular procedures, Prospective studies, Hemorrhage

## Abstract

**Purpose:**

To evaluate the early safety and technical feasibility of Amber 20 for peripheral vascular embolization in a prospective, multicenter, first-in-human stage I study.

**Materials and methods:**

This was a prospective, single-arm, multicenter, open-label first-in-human stage I trial conducted across 11 centers in Spain. Fifteen patients requiring peripheral embolization for various indications (including hemorrhage, aneurysm/pseudoaneurysm, endoleak, portal vein embolization, and hypervascular tumors) were enrolled. The primary endpoint was the rate of device-related serious adverse events (SAEs) within 24 h. Secondary endpoints included overall adverse events (AEs) and technical success, defined as complete vascular occlusion or devascularization on final angiography.

**Results:**

Fifteen patients (60% female; median age 56 years) underwent embolization using Amber 20. Technical success was achieved in 100% of cases (15/15). No device-related SAEs or AEs were reported within 24 h. Five patient (33%) experienced a mild adverse event (postembolization syndrome) related to the procedure. All adverse events resolved without sequelae. No device deficiencies were observed.

**Conclusion:**

In this first-in-human, multicenter study, Amber 20 demonstrated a favorable safety profile and high technical success rate for peripheral vascular embolization. These preliminary findings support its potential as an effective liquid embolic agent, warranting further evaluation in larger studies with longer follow-up (stage II).

**Graphical Abstract:**

**Figure:**
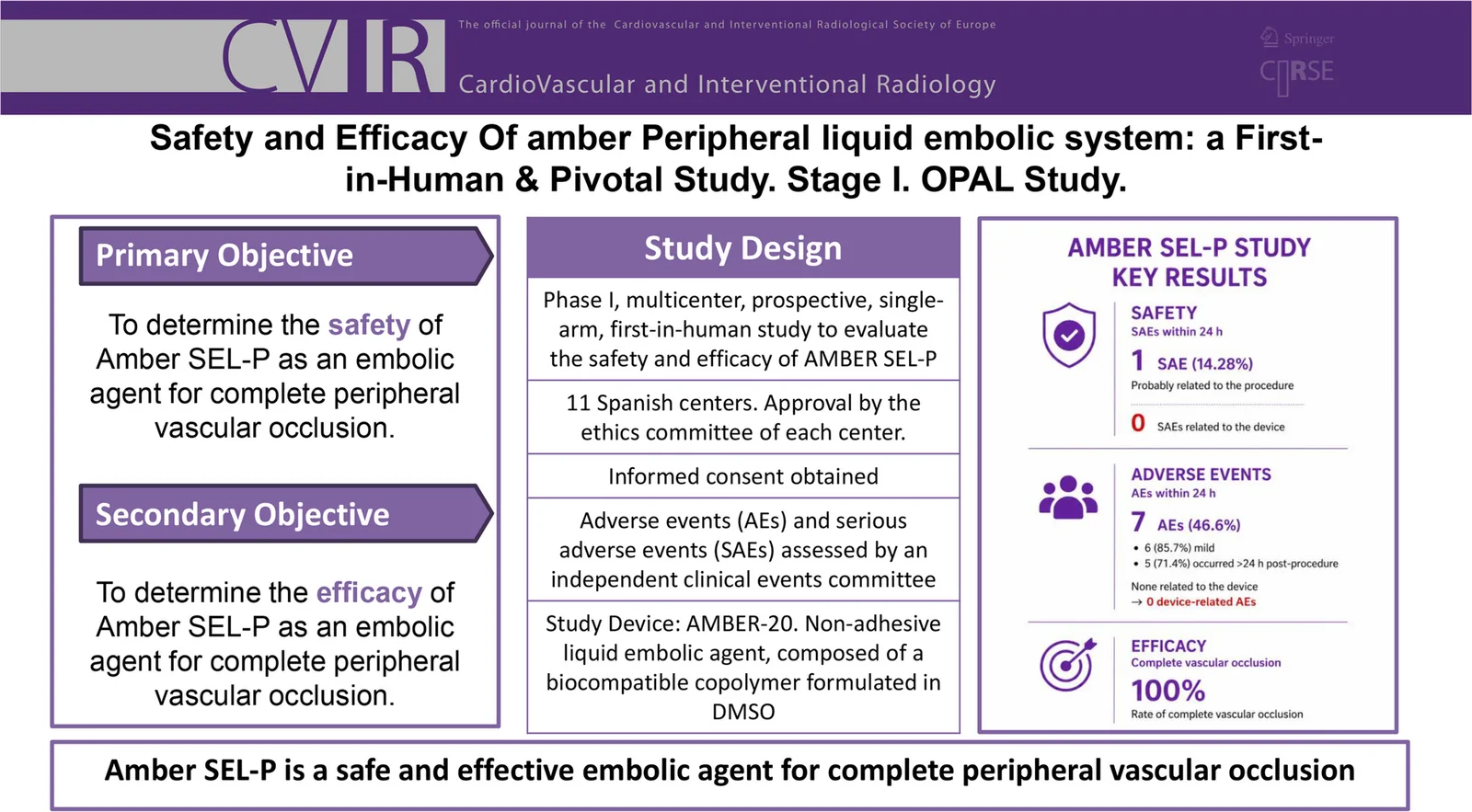

## Introduction

Transcatheter arterial and venous embolization is essential in managing various vascular conditions, including acute hemorrhage, tumors, arteriovenous malformations, venous varicosities, and endoleaks [1]. Advances in imaging, catheter technology, and embolic materials, ranging from autologous clots and Gelfoam to coils, glue, and microspheres [2], have significantly increased the complexity and effectiveness of these procedures.

Embolic agents can be classified by target vessel size [3]. For small vessels, available embolic devices include particles, liquid embolic agents (LEAs), and powders [4]. A critical consideration is that smaller agents are associated with a higher risk of organ ischemia [4]. LEAs have become a compelling strategy among smaller agents because of their versatility in several clinical indications.

LEAs can be classified into three groups according to their chemical characteristics: cyanoacrylates, ethylene vinyl alcohol copolymers, and ethiodol [5]. Each type entails its advantages and disadvantages. Some disadvantages include the need for non-ionic priming solutions with cyanoacrylates and the use of dimethyl sulfoxide (DMSO) as a solvent with ethylene vinyl alcohol copolymers. Ethylene vinyl alcohol copolymers need compatible microcatheters, lengthy preparation (shaking), and high cost [6]. Additionally, LEAs can cause significant imaging artifacts, especially on computed tomography (CT) [7]. A learning curve is required to handle these embolic agents effectively. However, the need for DMSO-compatible microcatheters and DMSO priming is shared by all DMSO-based agents, including Amber 20.

Desired improvements in the development of LEAs include minimizing some of these drawbacks by reducing imaging artifacts, incorporating suitable gelation mechanisms to prevent catheter occlusion, enhancing delivery control without the risk of fragmentation, and ensuring intrinsic radiopacity to eliminate the need for pre-mixing prior to injection [5]. Within this context, Amber 20 was developed to address some of these limitations, particularly prolonged preparation, fragmentation risk, and imaging artifacts, while retaining the DMSO-related requirements of this class of agents.

The aim of the OPAL-first in human/pivotal study stage I was to determine the safety of Amber, a new organic polymer system, for peripheral complete vascular occlusion. For this, OPAL was designed as a two-stage study; the first stage consisted of an initial feasibility study including 15 patients. The secondary outcome was to determine the efficacy of Amber as an organic polymer system, for peripheral complete vascular occlusion.

## Methods and materials

### Study design

A single-arm, prospective, multicenter, open-label, first-in-human study was conducted (ClinicalTrials.gov identifier: NCT06456125). The primary objective of this study was to evaluate the safety of Amber as an organic polymer system, for peripheral complete vascular occlusion. Secondary objective was to evaluate the efficacy of Amber as an organic polymer system, for peripheral complete vascular occlusion.

Investigators at 11 centers in Spain enrolled patients from May 2024 to November 2024 under a protocol approved by their institutional ethics committees. Patients provided written informed consent prior to participation in the study, and procedures were conducted in accordance with the Declaration of Helsinki and good clinical practices. The rate of any peri-procedure serious adverse events and adverse events during the peri-procedural period was assessed by an independent clinical events committee. Data was collected by the investigators using standardized web-based clinical report forms. Study sites were monitored for accuracy in database entry, and data was source-verified before database lock.

### Study eligibility

Patients aged ≥ 18 and < 95 years without a history of failed prior embolization and no allergy to Amber 20, DMSO, or contrast agent who require peripheral embolization due to varicose vein, type II endoleak, insufficient liver remnant requiring portal vein embolization (PVE) before liver resection, active arterial hemorrhage, aneurysm and/or pseudoaneurysm, pathologic organ, hypervascular tumor, and vascular anomalies (excluding the central nervous system and central circulatory system) were eligible for enrollment. Table 1 provides a detailed description of the inclusion and exclusion criteria according to the clinical condition.

**Table 1:** Inclusion and exclusion criteria

Inclusion criteria
Patients aged ≥ 18 and < 95 years
Varicose vein embolization
• Pelvic congestion syndrome: o Pelvic pain that often worsens as the day goes on and/or pain during intercourse or severe menstrual cramping and/or o Uterine venous engorgement and/or o Moderate or severe engorgement of the ovarian plexus and/or o Filling of the veins across the midline or filling of vulvar or thigh varicosities and/or o Reflux throughout the entire course of the ovarian vein
• Varicocele o Symptomatic varicocele > grade II and/or o Infertility or subfertility
• Varicose veins in patients with portal hypertension undergoing TIPS that require embolization
Type II endoleak
• Persistent type II endoleak and associated sac expansion > 5 mm after 6 months or 10 mm after 12 months
Insufficient liver remnant requiring portal vein embolization (PVE) before liver resection
• Predicted insufficient liver remnant after surgery (≤ 20% in a normal liver, ≤ 30% in liver with intermediate disease without cirrhosis, and ≤ 40% in liver with cirrhosis)
Active arterial hemorrhage and/or pseudoaneurysm
• Uncontrolled massive hemorrhage caused by tumor, trauma, or arteriovenous shunt formation (congenital or acquired)
• In case of bleeding or pseudoaneurysm up to 3 bleeding sites/pseudoaneurysms in the same organ or anatomic region
Pathologic organ (excluding brain):
• Non-functioning transplanted kidney or • Preoperative hip replacement or • Hypersplenism conditioning low platelet count or • Polycystic kidney disease or • Other
Hypervascular tumors
Vascular anomalies (excluding the central nervous system and central circulatory system)
Exclusion criteria
Patients with known hypersensitivity or allergy to Amber 20, DMSO solvent, or contrast agent
Individuals with significant impairment of hepatic and renal function, except for the following indications: non-functioning transplanted kidney and polycystic kidney disease in pathologic organ indication, varicose veins in patients with portal hypertension undergoing TIPS that require embolization, and portal vein embolization. The significant impairment hepatic and renal function is defined as: - Hepatic impaired function: class C Child–Pugh and/or bilirubin > 3 mg/dL - Renal impaired function: patients in stages 4 and 5 of CKD (glomerular filtration rate < 30 mL/min/1.73 m^2^) who are not in hemodialysis, or patients with acute renal failure
Previously failed embolization procedure, except for those treated with coils
Patient in whom according to the investigator criteria a complete vascular occlusion would not be feasible in a single procedure
Any condition that exposes the patient to a high risk for complications according to the investigator’s criteria (e.g., but not limited to, non-correctable coagulopathy, uncontrolled sepsis, underlying life-threatening condition)
Patients participating in another interventional study that has not completed its primary endpoint assessment
Pregnant or breastfeeding women, except emergent cases with potentially life-threatening active bleeding
Recurrent varicose vein embolization
Type II endoleak with: - High flow or reflux that cannot be prevented using coils or balloon microcatheter and/or - High risk of medullar ischemic damage
Active arterial bleeding and/or pseudo aneurysm with: - Severe hemodynamical instability (e.g., but not limited to, sustained hypotension [mean arterial pressure < 60 mmHg], tachycardia > 120 beats/minute, requirement of high doses of vasopressors) at the moment of the procedure and/or - Hb < 8 g/dL before the procedure and/or - In retroperitoneal hemorrhages or hemoptysis, identification of spinal or medullar vessels and/or - High risk of spinal cord ischemic damage
Central nervous system and central circulatory system vascular anomalies

### Study device

**Fig. 1:**
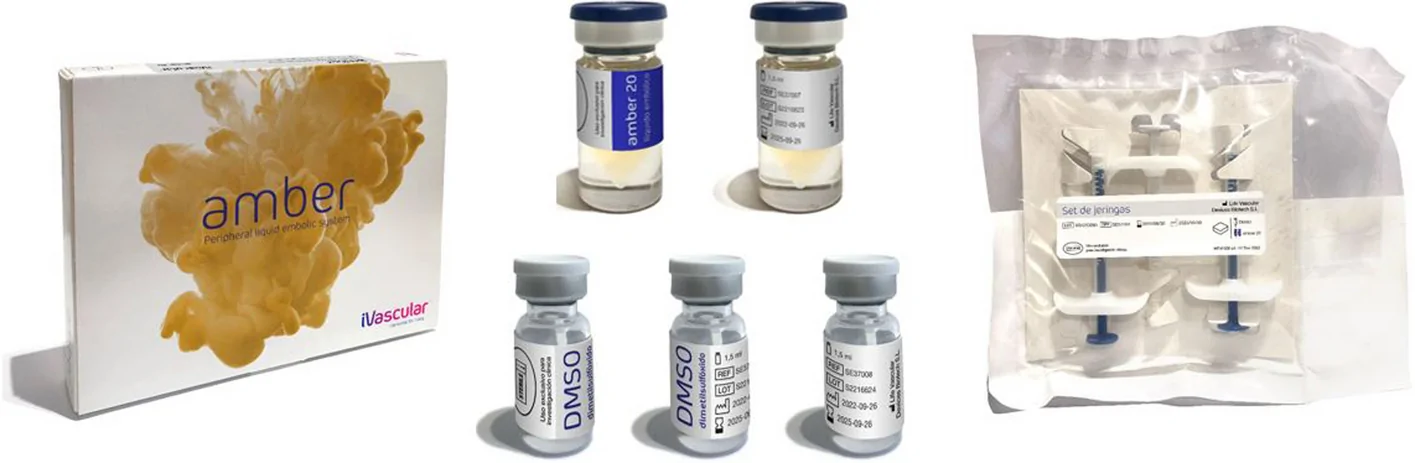
Components of Amber SEL-P. It contains 1 vial of Amber 20 (1.5 ml): non-adhesive embolic liquid composed of a polymer dissolved in DMSO (dimethyl sulfoxide); 1 vial of DMSO (1.5 ml): organic solvent used for the administration of Amber 20; and 1 syringe set: contains 2 syringes for administration of Amber 20 and 1 syringe for DMSO

Amber is a non-adhesive, dimethyl sulfoxide (DMSO)-based, tantalum-free embolic liquid system. This peripheral liquid embolic system offered in a single formulation that allows distal and deep injections contains the following: 1 vial of Amber 20 (1.5 ml), 1 vial of DMSO (1.5 ml), 2 syringes for administration of Amber 20, and 1 syringe for DMSO (Fig. 1). The copolymer is chemically bonded to an iodinated component, resulting in an iodinated polyacrylate that provides homogeneous radioscopic visualization. The iodine groups are covalently inserted into the polymer structure, creating a radiopaque, non-degradable, and stable polymer with the desired characteristics. The covalent bound iodine provides intrinsic radiopacity and may reduce CT artifacts compared with tantalum-based embolic agents, as suggested by preclinical studies. In addition, it allows the fluid to be used directly without prior preparation.

**Fig. 2:**
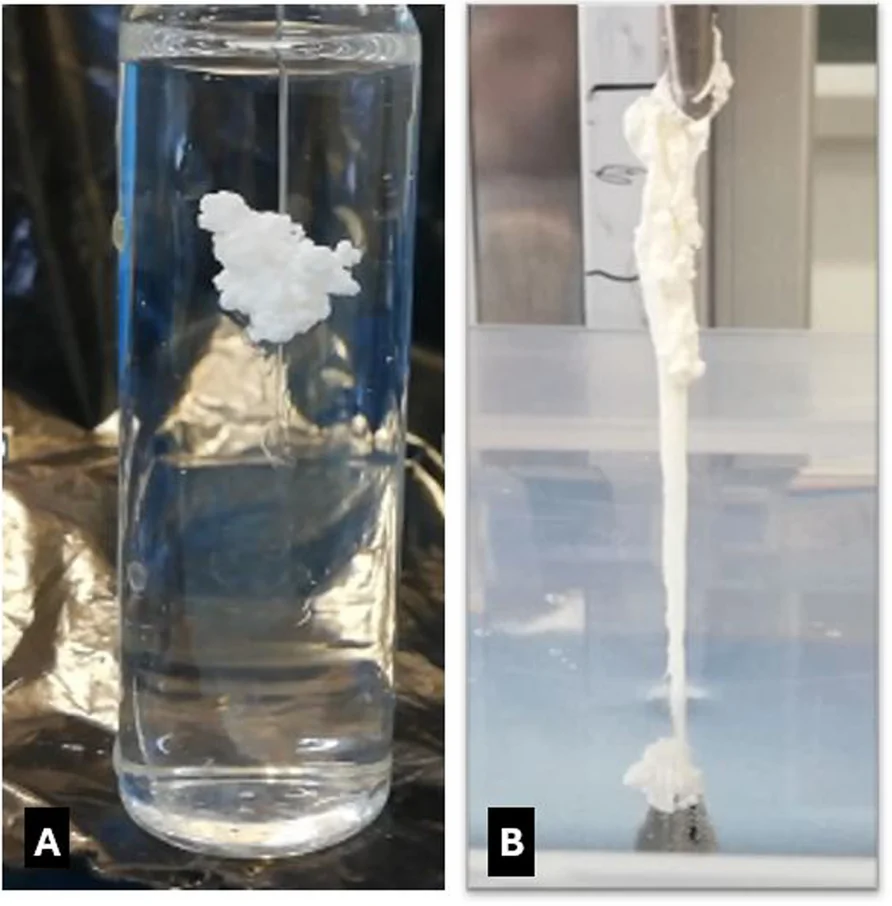
Study device: Amber SEL-P. Upon contact with the aqueous environment within the vasculature, an exchange of media (DMSO-aqueous medium) takes place, and a progressive precipitation process begins (**A**). Representative image illustrating the cohesive nature of Amber; the product maintains elasticity and resists fragmentation (**B**)

Amber 20 is administered by slow and controlled injection. Upon contact with the aqueous environment within the vasculature, an exchange of media (DMSO-aqueous medium) takes place, and a progressive precipitation process begins, allowing it to advance distally towards the interior of the vascular lesion until the polymeric embolus is formed (Fig. 2). The liquid solidifies after approximately 4 min, and since it does not adhere, it allows the physician to continue injecting in the starting position or to change position by moving the microcatheter until the embolization is completed, with a reduced risk of blockage and entrapment of the microcatheter.

DMSO is an organic solvent used to fill the empty space of the microcatheter that will be used during the injection of Amber 20, to avoid precipitation of the product. The microcatheters to be used during the procedure must have materials compatible with DMSO.

### Sample size calculation

The planned sample size of 70 participants referred to the complete two-stage OPAL investigation and not to the present stage I cohort. The first 15 patients were allocated to the stage I study, and the remaining 55 patients to the stage II study. The hypothesis was tested in the total study population (stage I + stage II). All calculations were performed using PASS 2023 software (v23) under a professional license. For the full study, a historical performance target was derived from a meta-analysis of 50 studies including 2204 patients, which reported a pooled technical success rate of 98.86% (95% CI, 97.11–99.56%). A clinically acceptable absolute reduction of 5 percentage points from the lower confidence limit was prespecified, resulting in a performance threshold of 92.1%. Assuming a true technical success rate of 98.86%, 63 evaluable participants would provide 80% power at a one-sided alpha level of 0.05; allowing for 10% attrition resulted in a planned total sample size of 70 participants. Therefore, Amber was considered non-inferior if the technical success rate was equal to or greater than 92.1%, leading to a non-inferiority margin of Δ = 92.1 − 98.86 = − 6.76. Thus, for the primary effectiveness endpoint, the statistical hypothesis test was defined as follows: H_0_: *π* − *π*_0_ ≤ − 0.676 (inferiority) versus H_1_: *π* − *π*_0_ > − 0.676 (non-inferiority), or equivalently: H_0_: *π* ≤ 0.921 (inferiority) versus H_1_: *π* > 0.921 (non-inferiority), where *π* denoted the technical success rate of Amber and *π*_0_ the reference technical success rate estimated in the meta-analysis (*π*_0_ = 0.9886).

The hypothesis was tested using a one-sided, one-sample *Z* test for proportions (normal approximation), with a type I error rate (*α*) of 0.05. The inclusion of 63 patients was sufficient to achieve a statistical power of 80% to assess the non-inferiority. Assuming an attrition rate of 10%, the sample size was increased to 70 patients.

### Patient characteristics and clinical variables

Clinical characteristics of patients were recorded, including demographic data, comorbidities, medical treatment, previous medical history, and clinical indications for embolization. Baseline imaging and laboratory data were collected prior to the procedure. Procedural data, encompassing the devices used, imaging modalities, and medications administered, were also documented. All collected variables are presented in Table 2.

**Table 2:** Study variables

Baseline visit
Demographic and anthropometric measurements • Date of birth, sex • Weight (kg) and height (cm) • Blood pressure (pressure gauge, mmHg) and cardiac rate (bpm)
Previous history • Drug allergies • Known diseases • Regular treatment • Any blood derivates transfusion
Imaging of the clinical indication for embolization • Varicose vein embolization: Doppler US and/or CT • Type II endoleak: Angio CT scan of the abdominal aorta or MRI • PVE: liver volume by CT or MRI • Hypervascular tumor: CT or MRI • Vascular anomalies: CT, MRI, and/or Doppler ultrasound scan • Active arterial bleeding, aneurysm, and pseudoaneurysm: CT or MRI • Pathologic organ: CT or MRI
Patient’s quality of life (EQ-5D)
Laboratories • Complete blood count • Renal and hepatic profile • Coagulation parameters • Spermiogram (varicocele)^a^
Pregnancy test (if applicable)
Visual Analog Scale (VAS) to evaluate pain improvement (varicose veins: varicocele and pelvic congestion syndrome)
Embolization procedure visit
Procedural data • Date and hour of beginning and ending • Type of sedation and route of access • Devices use • Medication use • Intraprocedural imaging (angiography) • Complications • Visual Analog Scale (VAS) to evaluate the pain at device injection • Mean volume of Amber 20 • Number of vials of Amber 20 • Mean volume of DMSO • Other embolic agent needed
Imaging • Hypervascular tumor: CT or MRI^a^
Laboratories, pre- and post-procedural (if available) • Complete blood count • Renal and hepatic profile • Coagulation parameters^a^
Post-procedural (discharge) imaging (if available)
Discharge medication
Adverse events • Any adverse event • Device-related • Access site related • Bleeding events o Any blood transfusion

^a^Not mandatory, only if available

### Endpoint definitions

Immediate technical success was defined as achievement of the prespecified angiographic treatment endpoint at the end of the index procedure. This endpoint was indication-specific: complete occlusion of the target bleeding vessel with cessation of extravasation for active hemorrhage; exclusion of the aneurysm or pseudoaneurysm from the circulation; complete occlusion of the intended portal venous branches for portal vein embolization; absence of residual filling of the treated endoleak cavity and feeding vessels for type II endoleak; and complete angiographic devascularization of the predefined target territory for hypervascular tumors or pathologic organ embolization.

Outcomes were assessed in terms of both safety and efficacy. The primary outcome was the 24-h rate of peri-procedural serious adverse events (SAE) related to Amber (device-related but not procedure-related). The secondary outcome was the 24-h rate of any peri-procedural adverse events (AE) related to the Amber (device-related but not procedure-related). The secondary safety endpoint was the incidence of adverse events within 24 h after embolization. The relationship of each event to the investigational device and to the embolization procedure was assessed separately rather than as mutually exclusive categories. Investigators performed the initial causality assessment, and all serious adverse events were independently adjudicated by the Clinical Events Committee. Device and procedural relationships were evaluated separately using previously specified categories based on temporal association, biological plausibility, the known consequences of embolization, angiographic evidence of non-target embolization (or device malfunction), and the presence of alternative clinical explanations. When a device contribution could not be confidently excluded, the event was classified conservatively as potentially device-related.

Efficacy was evaluated as the rate of complete vascular occlusion or correct devascularization of the treated lesion, as determined by angiographic assessment at the end of the procedure.

A SAE was defined as any adverse event that led to death, injury, or permanent impairment of a body structure or function. It was also considered a SAE if it resulted in a serious deterioration in the subject’s health, including a life-threatening illness or injury, permanent impairment of a body structure or function, in-patient hospitalization or prolonged hospitalization, or the need for medical or surgical intervention to prevent a life-threatening condition. Additionally, events leading to fetal distress, fetal death, or congenital abnormalities or birth defects were also classified as serious adverse events.

An AE was defined as any untoward medical occurrence, unintended disease or injury, or any untoward clinical signs (including abnormal laboratory findings) in subjects, users, or other individuals, regardless of whether it was related to the investigational device.

All the clinical investigation endpoints were defined according to the Society of Interventional Radiology (SIR) and Cardiovascular and Interventional Radiological Society of Europe (CIRSE) Quality Assurance Document and Standards for Classification of Complications definitions [8].

This stage I clinical trial will be considered successful if the rate of peri-procedural SAEs related to Amber (device-related but not procedure-related), adjudicated by an independent clinical events committee, is ≤ 20% (≤ 3 patients) within 24 h after the index procedure. The 20% threshold was preestablished in the study protocol as a pragmatic early safety stopping criterion for progression from stage I to stage II. It represented the maximum device-related serious adverse event rate considered clinically acceptable by the protocol steering group and the independent DSMB in this heterogeneous human population. With 15 participants in stage I, occurrence of four or more device-related SAEs would have exceeded this threshold and prevented automatic progression to stage II.

### Statistical analysis

Continuous variables were summarized using descriptive statistics including means and standard deviations if normally distributed or median with interquartile ranges for skewed distributions. Discrete variables were summarized using absolute and relative frequencies.

The total rate of peri-procedure SAEs related to Amber (device-related but not related to the procedure) at 24 h, adjudicated by an independent clinical events committee, will be computed. The total rate of complete vascular occlusion as defined by angiography at the end of the procedure will be computed.

## Results

Fifteen patients were consented and enrolled in the study between March 2024 and November 2024. Baseline patient demographics and medical histories are summarized in Table 3. Nine patients (60%) were female, with a median age of 56 years (IQR 34–74 years). The most common comorbidities were renal disease (*n* = 7, 46.7%), cardiovascular history (*n* = 6, 40%), and history of cancer (*n* = 6, 40%).

**Table 3:** Demographics, medical history, and current health status of study patients

	Total (*n* = 15)
Demographics	
Gender, female *n* (%)	9 (60)
Age, median (IQR)	56.0 (34.0, 74.0)
Weight (kg), median (IQR)	68 (46–96)
Height (cm), median (IQR)	164 (150–183)
Body mass index (kg/m), median (IQR)	24.9 (20.4–34.8)
Systolic blood pressure (mmHg), median (IQR)	130.0 (101.0, 159.0)
Diastolic blood pressure (mmHg), median (IQR)	76.0 (51.0, 99.0)
Heart rate (beats/min), median (IQR)	76.0 (55.0, 113.0)
Medical history and current health status	
Cardiovascular history (yes), *n* (%)	6 (40)
Bleeding disorder (yes), *n* (%)	0 (0)
Coagulopathy (yes), *n* (%)	1 (6.7)
Hematologic disease (yes), *n* (%)	1 (6.7)
Pulmonary disease (yes), *n* (%)	1 (6.7)
Renal disease (yes), *n* (%)	7 (46.7)
Gastrointestinal disease (yes), *n* (%)	5 (33.3)
Endocrine disease (yes), *n* (%)	1 (6.7)
Immunological disease (yes), *n* (%)	0 (0)
Diabetes (yes), *n* (%)	3 (20.0)
Connective tissue disorder (yes), *n* (%)	0 (0)
Cancer (yes), *n* (%)	6 (40)
History of allergy/hypersensitivity (yes), *n* (%)	2 (13.3)
Other relevant medical conditions (yes), *n* (%)	9 (60)
EQ5D VAS health status, median (range)	85.0 (30.0, 100.0)

**Fig. 3:**
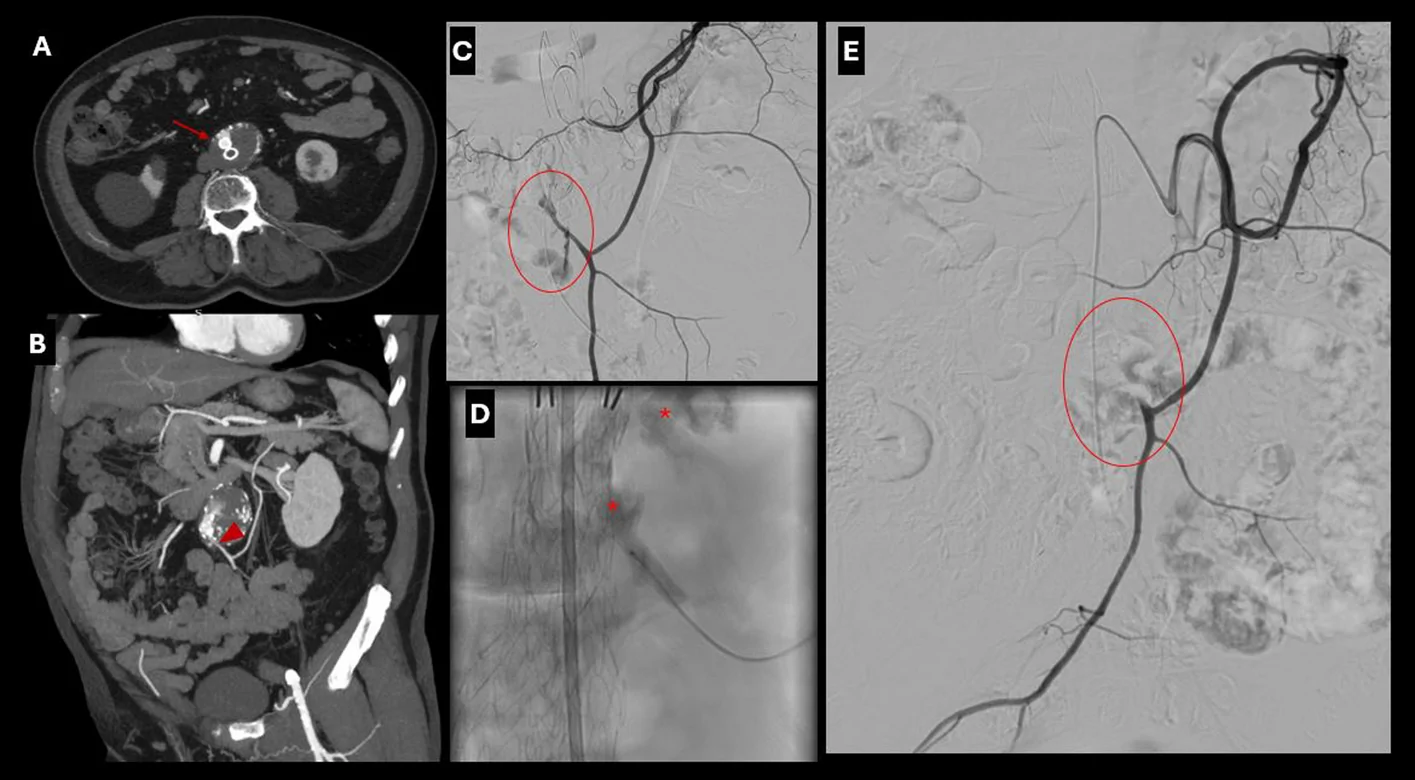
Type II endoleak. Male patient with a history of abdominal aortic aneurysm treated with EVAR. **A**, **B** AngioCT shows type II endoleak (arrow in **A**) originating from the inferior mesenteric artery (IMA) (origin of IMA, arrow head in **B**). **C** Selective arteriography from IMA shows endoleak (circle). **D** Distribution of Amber SEL-P within the sac (asterisks). **E** Control arteriography demonstrating resolution of the endoleak (circle)

**Fig. 4:**
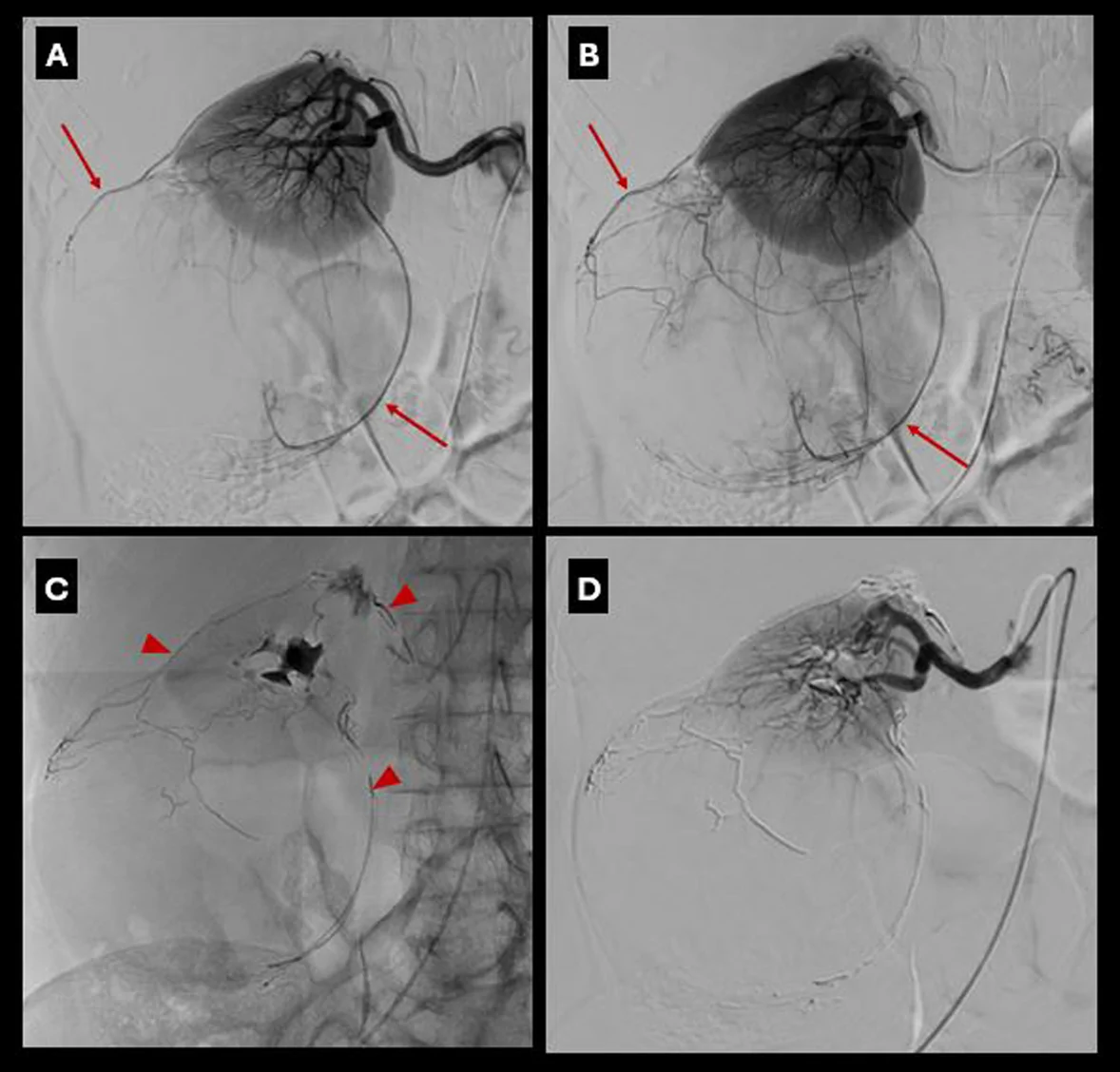
Hypervascular tumor, angiomyolipoma. **A**, **B** Initial arteriography showing a hypervascular lesion in the lower third of the right kidney, consistent with an angiomyolipoma (arrows). **C** Control image without subtraction and without contrast, demonstrating the vascular distribution of Amber (arrow head). **D** Final control arteriography showing devascularization of the lesion

Embolization indications included one case of type II endoleak (6.7%) (Fig. 3), one portal vein embolization (6.7%), four cases of active arterial hemorrhage, aneurysm, or pseudoaneurysm (26.7%), three cases of pathologic organ bleeding (20%), and six hypervascular tumors (40%) (Fig. 4). The preferred access route was femoral (*n* = 13, 86.7%). The number of embolization sites per procedure ranged from one artery (*n* = 10, 71.4%) to four arteries (*n* = 1, 7.1%). Most procedures required two vials per patient of Amber 20 treatment (*n* = 12, 80%). The prespecified indication-specific angiographic endpoint was achieved in all 15 procedures (100%; 95% CI, 78.2–100%). All embolization procedural data are summarized in Table 4.

**Table 4:** Embolization: procedural data

Embolization procedural data	Total (*n* = 15)
Procedure indication, *n* (%)	
Type II endoleak	1 (6.7)
Insufficient liver remnant requiring portal vein embolization (PVE) before liver resection	1 (6.7)
Active arterial hemorrhage/aneurysm and/or pseudoaneurysm	4 (26.7)
Pathologic organ	3 (20.0)
Hypervascular tumor	6 (40.0)
Device use, *n* (%)	
1	2 (13.3)
2	12 (80)
3	1 (6.7)
Route of access, *n* (%)	
Femoral right/left or both	13 (86.7)
Radial right/left or both	1 (6.7)
Other	1 (6.7)
Number of embolization sites, *n* (%)	
1	10 (71.4)
2	2 (14.3)
3	1 (7.1)
4	1 (7.1)
Missing	1
Pain at device injection (VAS), *n* (%)	
0	11 (78.6)
1	1 (7.1)
4	1 (7.1)
6	1 (7.1)
Missing	1
Complete vascular occlusion (yes), *n* (%)	15 (100)
Presence of intraprocedural complications (no), *n* (%)	15 (100)

Microcatheters were used in all patients in phase I (100%), with diameters of 1.9–2.7 Fr and lengths of 130–175 cm. The recorded models were Progreat (*n* = 11), Carnelian (*n* = 2), Xcepter XC (*n* = 1), and TruSelect (*n* = 1). Angiographic catheters were used in 13 patients, including Simmons, Cobra/Cobra 2, and multipurpose types, with diameters of 4–5 Fr and lengths of 65–125 cm. In addition, guiding catheters were used in 2 patients. Amber 20 was administered at an injection rate of 0.33 mL/min. The administered volume per procedure had a median of 1.5 mL (range: 0.2–12.0 mL) and a mean of 2.42 ± 2.87 mL. No other embolic agents were used in any of the procedures.

None of the AE was related to the device. Five patients (33%) experienced an adverse event, categorized as mild: postembolization syndrome with mild fever, which was not related to the device but likely related to the procedure, resulting in prolonged hospitalization. Of the seven total reported AEs, categorized as mild and moderated, two (28.6%) occurred within the first 24 h after the procedure, and none was related to the device. Three AEs (42.9%) were probably related to the procedure. All AEs (*n* = 7, 100%) resolved without sequelae. No device deficiency was reported. A full description of AEs is included in Table 5.

**Table 5:** Adverse events

ID	Indication	Time from procedure	Description	SAE	Device deficiency	Severity	Device related	Procedure related	Outcome
5	Hypervascular tumor	> 24 h	Postembolization syndrome	Yes	No	Mild	Not related	Probable	Resolved no sequalae
5	Hypervascular tumor	> 24 h	Scheduled hemicolectomy	No	No	Mild	Not related	Not related	Resolved no sequalae
7	Active arterial hemorrhage	< 24 h	Splenic ischemia, pain in shoulder	No	No	Mild	Not related	Probable	Ongoing
9	Hypervascular tumor	< 24 h	Possible reaction to drugs administrated during procedure	No	No	Mild	Not related	Not related	Resolved no sequalae
9	Hypervascular tumor	> 24 h	Gastrointestinal disorder	No	No	Mild	Not related	Not related	Resolved no sequalae
9	Hypervascular tumor	> 24 h	Fever	No	No	Moderate	Not related	Probable	Resolved no sequalae
13	Hypervascular tumor	> 24 h	Fever	No	No	Mild	Not related	Not related	Resolved no sequalae

## Discussion

Results from this prospective, multicenter, first-in-human, stage I trial demonstrated that Amber shows an encouraging early safety profile and technical feasibility. Amber is a safe and effective embolization system for peripheral vascular embolization. Complete vascular occlusion was achieved in all patients, with no device-related SAEs. The findings of this stage I trial support the safety profile of Amber for peripheral vascular embolization.

In this cohort, complete vascular occlusion or devascularization was achieved in all treated patients (*n* = 15, 100%), aligning with previously reported outcomes for other iodine-based embolic agents. For instance, Sapoval et al. [9] documented an immediate technical success rate of 98% (*n* = 49), defined as complete occlusion of the target vessel(s) on the final angiogram. Similarly, published analyses of liquid embolic agents consistently demonstrate very high immediate technical success rates, typically exceeding 95% [10].

Regarding safety, no device-related SAEs or AEs were observed. A single mild SAE, likely related to the procedure rather than the device, was reported: postembolization syndrome with low-grade fever, which resulted in prolonged hospitalization. Postembolization syndrome is a well-recognized clinical condition following embolization procedures, typically resulting from the inflammatory and ischemic effects of embolization on the treated tissues. Symptoms usually appear within hours to a few days post-procedure and may include fever (commonly low-grade but occasionally > 38 °C), abdominal or localized pain at the embolized site, nausea, vomiting, fatigue or malaise, and anorexia [11]. In comparison, Sapoval et al. reported three major device-related complications in two patients, adjudicated by an independent Critical Events Committee, all attributed to non-target embolization [9].

In our experience, the ready-to-use formulation of Amber offered a practical advantage by reducing preparation time, as it does not require the pre-injection agitation necessary with other tantalum-based copolymers. This characteristic may be particularly relevant in scenarios demanding rapid intervention, such as active arterial bleeding. In this stage I trial, complete vascular occlusion was achieved in all patients presenting with active hemorrhage, aneurysm, or pseudoaneurysm (*n* = 4, 26.7%) without any SAEs related to the device. These findings suggest that Amber could represent a valuable option in urgent endovascular settings; however, the small sample size and early-phase nature of this study limit the generalizability of our results.

Its bonded iodine-based composition ensures stable radiopacity, unlike tantalum-based embolic agents, which are prone to sedimentation with time and subsequent loss of radiopacity [12]. These properties of Amber may contribute to improved procedural efficiency and operator confidence.

A preclinical study suggested that iodine-based embolic agents may produce fewer CT artifacts than tantalum-containing copolymers. However, CT artifacts were not assessed qualitatively or quantitatively in the present work; therefore, no conclusion regarding the comparative artifact profile of Amber 20 can be drawn from these clinical data [13]. The absence of CT imaging artifacts due to the lack of tantalum powder in the device is an advantage especially in cases in which CT follow-up is important, for example, in the follow-up of type II endoleaks, representing a major advantage over other liquid embolic agents (Fig. 3).

This prospective, multicenter, first-in-human stage I trial evaluating the safety and efficacy of Amber has inherent limitations, including a limited sample size, heterogeneity of clinical indications, and the absence of long-term follow-up data. A major limitation is that the 24-h primary safety window, selected to capture immediate device- or procedure-related events and support early DSMB review, may not detect delayed complications such as non-target embolization, ischemic injury, or inflammatory reactions. To address these limitations and further characterize the safety and efficacy profile of Amber as a liquid embolic agent system for complete peripheral vascular occlusion, a stage II trial with a 6-month follow-up is currently underway.

In conclusion, this stage I study provides preliminary evidence of the early safety and technical feasibility of Amber 20. The observed immediate angiographic occlusion rate is encouraging but should be interpreted descriptively and requires confirmation in the larger stage II cohort and in indication-specific comparative studies.

## Data Availability

Individual participant data from the OPAL study are not publicly available. Requests for access to de-identified data may be submitted to the corresponding author and are subject to approval by the study sponsor.
